# Systemic Importance of China’s Financial Institutions: A Jump Volatility Spillover Network Review

**DOI:** 10.3390/e22050588

**Published:** 2020-05-24

**Authors:** Xin Yang, Xian Zhao, Xu Gong, Xiaoguang Yang, Chuangxia Huang

**Affiliations:** 1 School of Mathematics and Statistics, Changsha University of Science and Technology, Changsha 410114, China; yangxintaoyuan@csust.edu.cn (X.Y.); zhaoxian1714@163.com (X.Z.); 2 Hunan Provincial Key Laboratory of Mathematical Modeling and Analysis in Engineering, Changsha University of Science and Technology, Changsha 410114, China; 3 School of Management, China Institute for Studies in Energy Policy, Xiamen University, Xiamen 361005, China; xugong@xmu.edu.cn; 4 Academy of Mathematics and Systems Science, Chinese Academy of Sciences, Beijing 100190, China; xgyang@iss.ac.cn

**Keywords:** financial institution, complex network, jump volatility, entropy weight TOPSIS

## Abstract

The investigation of the systemic importance of financial institutions (SIFIs) has become a hot topic in the field of financial risk management. By making full use of 5-min high-frequency data, and with the help of the method of entropy weight technique for order preference by similarities to ideal solution (TOPSIS), this paper builds jump volatility spillover network of China’s financial institutions to measure the SIFIs. We find that: (i) state-owned depositories and large insurers display SIFIs according to the score of entropy weight TOPSIS; (ii) total connectedness of financial institution networks reveal that Industrial Bank, Ping An Bank and Pacific Securities play an important role when financial market is under pressure, especially during the subprime crisis, the European sovereign debt crisis and China’s stock market disaster; (iii) an interesting finding shows that some small financial institutions are also SIFIs during the financial crisis and cannot be ignored.

## 1. Introduction

With the development of economic globalization, the financial system has become more and more closely interconnected by investment networks, debtor–creditor and trade contacts [1,2,3,4]. Financial institutions such as depositories, broker-dealers and insurance companies permeate each other by related business and display significant complex network properties [5,6,7]. The failure of several financial institutions may lead to a severe economic crisis [8,9,10]. One of the typical examples is the global financial crisis triggered by the collapse of Lehman Brothers in 2008 [11,12,13]. Therefore, how to accurately evaluate the systemic importance of financial institutions (SIFIs) so as to provide early warning and deal with the crisis effectively has become an emergent work [14,15,16].

Usually, there are three ways to measure the SIFIs. The first way is to employ Pearson correlation coefficient to calculate the financial institutions’ default probabilities [17,18,19]. Pearson correlation coefficient ignores the heterogeneity of financial data at different times [20]. Adopting a tail-dependence method to measure the systemic risk contributions between financial institutions is the second method. Girardi and Ergün (2013) [21] used the conditional value-at-risk (CoVaR) method to estimate systemic risk of each financial institution. Acharya et al. (2017) [22] employed the systemic expected shortfall (SES) model to calculate financial institution’s losses by considering its leverage. Wang et al. (2019) [23] proposed CSRISK model to investigate financial institutions’ capital shortfall under the market crash. The above two methods are based on the local correlation and disregard the interlinked among the financial institutions, which may underrate systemic risk contribution [24]. The latest financial crisis manifests that intricate connections among financial markets can spread risk [25,26,27,28,29]. Using the complex network theory to research SIFIs comes up to the third method. Billio et al. (2012) and Gong et al. (2019) [30,31] applied Granger causality model to build financial institution network and utilized the out degree to compute the total connectedness. Hautsch et al. (2014) [24] proposed VaR model to set up financial institution network and adopted systemic risk betas to investigate the systemic risk contribution. Härdle et al. (2016) and Wang et al. (2018) [32,33] adopted CoVaR model to construct financial institution network and used the index of out degree to measure the system risk contribution.

Most of the literature evaluates the SIFIs by out degree [31,32,33], which can reveal the range of risk contagion but restrict to the local information of the network [34,35]. Recently, considering financial institutions have the characteristics of deeper risk contagion extent, higher risk contagion efficiency and greater risk contagion degree after the outbreak of financial risk, some other indicators such as, clustering coefficient [36], closeness centrality [37] and Leaderrank value [38] are also applied to measure the SIFIs. Although most of the above indicators have been investigated extensively and many findings on SIFIs have been reported recently, which only reflect one characteristic of the network [36,37,38]. A comprehensive evaluation with respect to the entire network appears to be very few. Such studies are however essential to accurately evaluate SIFIs in practice. To deal with this issue, combining four indicators (out degree [32], clustering coefficient [36], closeness centrality [37] and Leaderrank value [38]) and assigning different weights to each indicator may give a better evaluation. However, the selection of weight is often based on the subjective experience of researchers, rather than sufficient scientific support, which may lead to inaccurate evaluation results. As we all known, entropy weight technique for ioder preference by similarities to ideal solution (TOPSIS) is a multiple criteria decision making method, and it bases on the conception that the selected alternative should have the shortest distance from the positive ideal solution and the farthest distance from the negative ideal solution. Entropy weight TOPSIS has been proved to be a good method in strategic decision making and successfully applied in some fields, such as coal mine safety [39], multinational consumer electronics company [40] and transport [41]. Therefore, it seems that adopting entropy weight TOPSIS to comprehensively assess the SIFIs might be a better choice.

It should be pointed out that the all above mentioned literatures on measuring SIFIs have been greatly limited to low-frequency data. The low-frequency data with daily, weekly, monthly, quarterly or annual sampling frequency can not accurately measure the whole-day volatility information [42]. Nowadays, more and more scholars have realized that the high-frequency data with the frequency of hours, minutes or even shorter includes the rich information of asset price, and it has been intensively studied in applied finance risk management [43,44,45,46]. On the other hand, with the unexpected changes of macroeconomic conditions, international events and economic policy in recent years, financial markets are increasingly volatile [47]. Some researches detected jump volatility in the volatile process of financial assets based on high-frequency data [48]. For example, Wright and Zhou (2007) [49] found that jump volatility can explain much of the countercyclical movements in bond risk premium. Zhang et al. (2016) [50] found that jump volatility is an important component of Dow Jones Industrial Average stocks’ volatility. Audrino and Hu (2016) [51] found that jump volatility can improve the forecast of S&P 500’s volatility.

The jump volatility depicts an infrequent but a sharp change of asset price, and it can better describe violent volatility of financial market than continuous volatility [52]. Measuring SIFIs associated with jump volatility spillover network and high-frequency data has not been reported yet and it still remains a challenging problem. Motivated by the above discussions, in this paper, we aim to employ high-frequency data of China’s financial institutions to construct jump volatility spillover network, and then utilize entropy weight TOPSIS to comprehensively assess the SIFIs. The innovations of this paper are as follows:

(1) Many scholars investigated the jump volatility of a single financial asset on its price fluctuation from the perspective of prediction. We first propose Granger-causality test to identify the jump volatility spillover among financial institutions.
(2) Financial markets are extremely volatile, and the low-frequency data might lose a lot of important information. By employing 5-min high-frequency data, we establish the jump volatility spillover network, which can capture the jump volatility spillover among financial institutions.
(3) We use entropy weight TOPSIS rather than a single indicator to comprehensively assess the SIFIs.

The reminder of this paper is arranged as follows. In Section 2, we introduce the methodology. In Section 3, we present the data. In Section 4, we give an empirical analysis. Finally, we make conclusions and discuss our findings in Section 5.

## 2. Methodology

In this section, we introduce the method of network construction and the indicator for assessing the SIFIs. Specifically, in Section 2.1, we use Granger causality test to build the network, which reflects statistically significant relations between jump volatility spillover of financial institutions. In Section 2.2, out degree, clustering coefficient, closeness centrality and leaderrank algorithm are employed to evaluate the SIFIs, respectively. In Section 2.3, by the method of entropy weight TOPSIS, we integrate the above four indicators into a comprehensive indicator to measure the SIFIs.

### 2.1. Network Construction

We establish jump volatility spillover network of financial institution according to the following three steps, where each financial institution represents a network node, and each pair of the financial institution is connected with an edge calculated by Granger-causality test.

In the first step, we employ Andersen et al. (2007, 2012) tests to extract jump volatility of financial institutions. We suppose that the logarithmic price of a financial institution ($p_{t}=\ln P_{t}$) within the trading day obeys a standard jump diffusion process:

(1) $$\begin{array}{c} dp_{t}=\mu_{t}dt+\sigma_{t}dW_{t}+\kappa_{t}dq_{t},\;0⩽t⩽T, \end{array}$$

where $\mu_{t}$ denotes the drift term, which includes a continuous volatility sample path; $\sigma_{t}$ represents a strictly positive stochastic volatility process; $W_{t}$ stands for a standard Brownian motion; $\kappa_{t}dq_{t}$ is the pure jump component.

Meanwhile, the logarithmic return volatility can be expressed as quadratic volatility, which contains jump volatility rather than unbiased estimator of integrated volatility:

(2) $$QV_{t}=\int_{t-1}^{t}\sigma_{s}^{2}ds+\sum_{t-1<s\leq t}k_{s}^{2}$$

where $\int_{t-1}^{t}\sigma_{s}^{2}$ represents the continuous volatility, and $\sum_{t-1<s\leq t}k_{s}^{2}$ stands for intra-day jump volatility.

Since the quadratic volatility can not be gained directly, this paper employs the estimated realized volatility $RV_{t}$ to replace it based on Andersen et al. (2012) [53]:

(3) $$RV_{t}=\sum_{i=1}^{M}r_{t,i}^{2},$$

where $r_{t,i}=\left(\ln P_{t,i}-\ln P_{t-1,i}\right)\times 100$, $P_{t,i}$ denotes the closing price of financial institution *i* at time *t*, and *M* = 48 represents the daily trading frequency.

In addition, when M→∞, $\int_{t-1}^{t}\sigma_{s}^{2}ds$ can be calculated by the realized bipower volatility $MedRV_{t}$ based on Barndorff-Nielse et al. (2004) [54,55].

(4) $$MedRV_{t}=\frac{\pi }{6-4\sqrt{3}-\pi }\left(\frac{M}{M-2}\right)\sum_{i=2}^{M-1}\mathrm{Med}{\left(\left|r_{t,i-1|}\right|r_{t,i}\left|\right|r_{r,i+1|}\right)}^{2},$$

If there is no jump in the price of financial institutions, the difference between realized volatility and bipower volatility is 0. Otherwise, Z-statistic is adopted to identify jump volatility [54]:

(5) $$Z_{t}=\frac{\left(RV_{t}-MedRV_{t}\right)RV_{t}^{-1}}{\sqrt{\left(\mu_{1}^{-4}+2\mu_{1}^{-2}-5\right)\frac{1}{M}\max\left(1,\frac{medRTQ_{t}}{medRV_{t}^{2}}\right)}}\to N\left(0,1\right),$$

where $\mu_{1}=\sqrt{2/\pi }$, and $\mathrm{med}RTQ_{t}=\frac{3\pi M}{9\pi +72-52\sqrt{3}}\left(\frac{M}{M-2}\right)\sum_{i=2}^{M-1}\mathrm{Med}{\left(\left|r_{t,i-1|}\right|r_{t,i}\left|\right|r_{r,i+1|}\right)}^{4}$, which stands for realized tri-power quarticity.

Based on Z statistics, we can obtain realized jump volatility:

(6) $$J_{t}^{d}=I\left\{Z_{t}>\Phi_{t}\right\}\left(RV_{t}-MedRV_{t}\right),$$

where I(·) is an indicator function, and α chooses as 0.95 (see Andersen et al., 2007 [56]).

In the second step, after extracting the jump volatility of a single financial institution, we investigate whether there is jump volatility spillover between financial institutions according to Granger-causality test [57]. If the *p* values of Granger-causality test are smaller than the critical values under the 5% significance level [58], there exists causality relationships between financial institutions.

In the last step, we construct a Granger-causality jump volatility spillover network of financial institutions. And the network can be represented by an adjacency matrix AD:

(7) $$\begin{array}{c} AD=\left(V,E\right)=\left[\begin{array}{cccccc} 0 & \cdots & \cdots & AD_{1j} & \cdots & AD_{1n}\\ \vdots & \ddots & \ddots & \vdots & \ddots & \vdots \\ AD_{i1} & \ddots & \ddots & AD_{ij} & \ddots & AD_{in}\\ \vdots & \ddots & \ddots & \vdots & \ddots & \vdots \\ \vdots & \ddots & \ddots & \vdots & \ddots & \vdots \\ AD_{n1} & \cdots & \cdots & AD_{nj} & \cdots & 0 \end{array}\right] \end{array}$$

where *V* is nodes set and *E* is the edge set. *n* is the number of financial institutions. $AD_{ij}$ is defined as follow:

(8) $$\begin{array}{c} AD_{ij}=\left\{\begin{array}{cc} 1 & i\;Granger\;causes\;j\;significantly\\ 0 & i\;doesn^{\prime }t\;Granger\;causes\;j \end{array}\right. \end{array}$$

### 2.2. Indicator for Assessing the Systemic Importance of Financial Institutions

There are a growing number of indicators to evaluate SIFIs. Taking into account financial institutions have the characteristics of wider risk contagion range, deeper risk contagion extent, higher risk contagion efficiency and greater risk contagion degree, we choose out degree, clustering coefficient, closeness centrality and leaderrank value to assess the SIFIs, respectively. And more and more scholars use these four indicators to study the SIFIs [33,36,37,38].

#### 2.2.1. Out Degree

Out degree (OD) calculates the number of edges that node *i* point to other nodes. It is used to measure the risk contagion range [36]. When the risk occurs, it will directly transfer the risk to the connected nodes. The higher the out degree of nodes, the wider the range of risk transmission. The expression of out degree is as follows:

(9) $$\begin{array}{c} OD_{out}\left(i\right)=\sum_{j=1}^{n}AD_{ij}, \end{array}$$

where $AD_{ij}$ stands for the adjacency matrix of financial institution network.

#### 2.2.2. Clustering Coefficient

The clustering coefficient (*C*) measures the degree of interconnection between the neighbors of a node in the graph. If one node owes high clustering coefficient, the risk may spread to their neighbor nodes when one financial institution fluctuates. Furthermore, the interconnectedness of neighbor nodes will cause risk contagion again and aggravate the risk contagion extent of the whole financial institutions [36]. Therefore, we employ clustering coefficient as the risk contagion extent of each financial institution, and it is computed as follows:

(10) $$\begin{array}{c} C_{i}=m_{i}/\alpha_{i}\left(\alpha_{i}-1\right), \end{array}$$

where $\alpha_{i}\left(\alpha_{i}-1\right)$ represents the maximum number of possible edges of financial institution *i*, and $m_{i}$ stands for the actual number of existing edges.

#### 2.2.3. Closeness Centrality

Closeness centrality (CC) quantifies how close a node is to all other nodes in the financial institution network. The closeness centrality of a node is inversely proportional to the average shortest path distance from one node to any other nodes in the network. The larger value of the closeness centrality of a node, the faster the risk will be transferred from one node to any other nodes. Hence, the closeness centrality can depict how efficiently each node transmits risk to all other nodes [37], and it is expressed as follows:

(11) $$\begin{array}{c} CC_{out}\left(i\right)=\sum_{j=1,j\neq i}^{N}2^{-d_{ij}}, \end{array}$$

where $d_{ij}$ is the shortest distance *i* to *j*.

#### 2.2.4. Leaderrank Algorithm

LeaderRank (LR) algorithm is a method to identify key nodes in a complex network. The basic idea of the algorithm is as follows. We add a new node (called ground node) and connect it to all others by bidirectional edges for a directed network with *M* nodes and *N* edges. The new network is strongly linked, which owes M+1 nodes and N+2M edges. Matrix $A=(a_{ij})$ depicts the connectivity of the network. If $AD_{ij}=1$, which means that node *i* can pass financial risk to node *j*. The LR gives a score to each node, where score denotes the SIFIs. Scores are assign by $LR_{g}\left(0\right)=0$ for ground node and $LR_{i}\left(0\right)=1$ for other nodes. Thus, scores are updated by

(12) $$\begin{array}{c} LR_{i}\left(t\right)=\sum_{j=1}^{M+1}\frac{a_{ji}}{OD_{out}\left(j\right)}LR_{i}\left(t-1\right), \end{array}$$

where $OD_{out}\left(j\right)$ is out degree.

After *t* iterations, the LR values of all nodes are stable. At this time, the ground node score is averagely distributed to each network node. Consequently, the final score of the network node reflects its cumulative risk ability. The higher the score, the stronger the cumulative risk degree of the node [38].

### 2.3. Entropy Weight TOPSIS

Measuring risk contagion of financial institutions from different indicators may lead to inconsistent results. Therefore, the construction of risk contagion composite index of financial institutions is an essential step in this paper. We adopt entropy weight TOPSIS to evaluate SIFIs. It can avoid the subjectivity of weight selection and make full use of the sample data [39,40,41].

The entropy of each indicator is calculated as below:

(13) $$e_{j}=-\frac{1}{\ln N}\sum_{i=1}^{N}p_{ij}\ln p_{ij},p_{ij}=\frac{x_{ij}}{\sum_{i=1}^{N}x_{ij}^{2}},$$

where j=1,…N; i=1,…n; *N* = 24; *n* = 4, $x_{ij}$ denotes the *j*th indicator value of the *i*th financial institution of the initial matrix *X*; $p_{ij}$ stands for the *j*th normalized indicator value of the *i*th normalized matrix *P*.

The weight ($w_{j}$) can be calculated as follows:

(14) $$w_{j}=\frac{1-e_{j}}{N-\sum_{i=1}^{n}e_{j}},j=1,2,\cdots ,N.$$

Then, the TOPSIS method ranks financial institutions based on their relative proximities, and the positive ideal solution and the negative ideal solution. The distance of each indicator from $D_{i}^{+}$ and $D_{i}^{-}$ can be calculated as the following:

(15) $$D_{i}^{+}=\sqrt{\sum_{j=1}^{N}{\left(S_{ij}-S_{i}^{+}\right)}^{2}},j=1,2,\cdots ,N,$$

(16) $$D_{i}^{-}=\sqrt{\sum_{j=1}^{N}{\left(S_{ij}-S_{j}^{-}\right)}^{2}},j=1,2,\cdots ,N,$$

where $S_{ij}=w_{j}p_{ij}$, $S_{i}^{+}=\max_{1\leq j\leq n}\left(S_{ij}\right)$, and $S_{i}^{-}=\min_{1\leq j\leq n}\left(S_{ij}\right)$.

The relative proximity $K_{i}$, which is regarded as score of each financial institution, can be computed as follows:

(17) $$K_{i}=D_{i}^{-}/\left(D_{i}^{-}+D_{i}^{+}\right),\;j=1,2,\cdots ,N.$$

Finally, in order to measure ability of risk contagion, we use $K_{i}$ to rank the SIFIs.

## 3. Data

We select 24 listed financial institutions from 2008 to 2018 in China, similar sample selection can be found in Wang et al. (2018) [33]. We choose 2008 as the starting date due to several important financial institutions do not go public until 2007, such as the China Construction Bank. We divide listed financial institutions into three sectors: (1) depositories, (2) insurance companies and (3) broker dealers. The data are available from Wind Financial dataset, and the descriptive statistics of 5-min high-frequency closing price data of financial institutions are shown in Table 1.

## 4. Empirical Analysis

### 4.1. Jump Volatility Spillover Network Construction of Financial Institution

**Figure 1:**
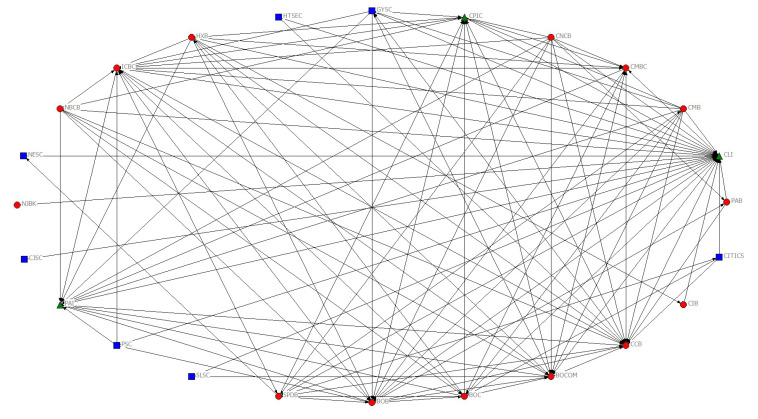
Jump volatility spillover network of financial institution. Note: Nodes (financial institutions) from the same sector are signed as the same shape and color. Depositories, broker dealer, and insurances are labelled as red circle, blue square, and green triangle, respectively.

The sampling frequency selection of intraday high-frequency data is very important for jump volatility measurement. The low sampling frequency may not fully express the jump volatility information, while high sampling frequency can cause micro structural noise. According to Haugom et al. (2014) [59], Gong and Lin (2018) [46] and Wen et al. (2019) [55], this paper first adopts 5-min high-frequency closing price data of financial institutions to compute jump volatility based on Equations (1)–(9). Then, using Granger causality test to investigate jump volatility spillover relations among financial institutions, we can obtain the *p* value of a 24 × 24 matrix. This paper chooses a threshold of 0.05 according to Jiang et al. (2017) [58]. Finally, the financial institution network gets a total of 24 nodes and 137 edges, and the results are illustrated by Figure 1.

### 4.2. Assessing the Systemic Importance of Financial Institutions

The research on the systemic importance of financial institutions (SIFIs) has become a hot topic in financial risk management. In this section, we choose out degree, clustering coefficient, closeness centrality and leaderrank value to measure the SIFIs. The results are shown in Table 2.

Table 2 displays four dimensions of risk contagion measurement. (1) In terms of risk contagion range, the larger the out degree value, the wider risk contagion range of financial institution. We can find that CMB, BOC and CNCB are all from depository sector, with the highest out degree value of 10. One possible reason is that China’s financial system is a depository-led system. As claimed by the annual reports of CBRC, CSRC, and CIRC in 2019, the total assets of depository, broke-dealer and insurance sectors were 261.4 trillion Yuan, 6.2 trillion Yuan and 18.3 trillion Yuan, respectively. This indicates that the depository sector size is 42 times larger than the broke-dealer sector, or 14 times larger than the insurance sector. (2) In terms of risk contagion extent, the larger the clustering coefficient value, the greater risk contagion extent of financial institution. We can see that SLSC and HTSEC, which are all from broke-dealer sector, have the highest clustering coefficient value. The results show that broke-dealer sector’s risk contagion ability can not be neglected in China’s financial system. (3) In terms of risk contagion efficiency, the larger the closeness centrality value, the faster the risk will be transferred from one financial institution to any other financial institutions. We can discover that CJSC and PAI have the highest closeness centrality value, indicating that some broke-dealers and insurances have gradually become important departments in China’s banking-led financial system. (4) In terms of risk contagion degree, the larger the leaderrank value, the higher the risk contagion degree. The risk contagion degree of financial institutions manifests a hierarchical feature, i.e., the greatest degree of risk contagion is insurance sector, followed by broke-dealer sector, and depository sector have the lowest risk contagion degree.

The results of the above four dimensions are inconsistent in measuring the SIFIs. In order to comprehensively assess the SIFIs, this paper proposes entropy weight TOPSIS (EWTOPSIS) to obtain the weight of each indicator. As a result, we gain the weight of out degree, clustering coefficient, closeness centrality and leaderrank algorithm by 0.2807, 0.2499, 0.0401 and 0.4293, respectively.

Table 3 shows the score of SIFIs computed by EWTOPSIS in the whole period. We can find that: (1) CLI is the most SIFIs. This may be related to the deregulation reform of the China’s insurance sector in 2014. China’s state council issue that the insurance depth will reach 5% and the insurance density will reach 3500 yuan/person by 2020. (2) CCB, BOB, ICBC and BOCOM are deem as more systemically important financial institutions. Because most of them come from state-owned depositories, which dominate China’s depository sector about 45% of the lending business in 2018. (3) CJSC and NJBK have a relatively low score, which imply that the impact of some small financial institutions on the financial system can be neglected.

Compared with the traditional evaluation SIFIs methods such as Equal weight [38], principal component analysis (PCA) and TOPSIS method [60], we will show our proposed EWTOPSIS method has obvious advantages. Just as reported by Sandoval (2014) [61], Wang et al. (2018) [33] and Wang et al. (2019) [62], market capitalization as a financial indicator could reflect the market influence of financial institutions. We calculate the correlation between each index (depending on different methods) and market capitalization, and the results can be presented as Table 4. It is easy to see that the EWTOPSIS is most effective method to measure the SIFIs because of the correlation between EWTOPSIS and market capitalization is the largest at a significant level of 1%.

### 4.3. Assessing the Dynamic Systemic Importance of Financial Institutions

As we all know, the financial markets are complex dynamic systems. The jump volatility spillover between financial institutions is time-varying. Thus, we employ time-varying Granger causality test to build dynamic jump volatility spillover of financial institution networks [63]. 2677 financial institution networks are obtained.

**Figure 2:**
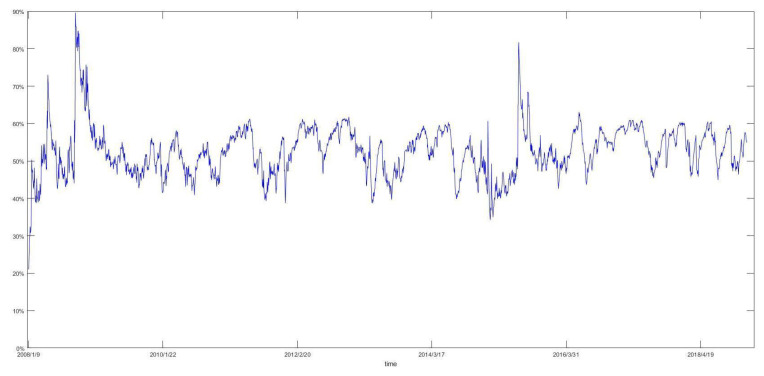
The number of total linkages of the jump volatility spillover network as a percentage of all possible linkages over time.

Figure 2 exhibits the evolution of the number of total linkages as a percentage of all possible linkages (TP). We can discover that it has three prominent cycles based on high TP values. The first cycle started at January 2008 and ended until April 2008, which was in the period of the subprime crisis. During this time, the CSI 300 Index decreased by 661 points (almost 17%) from 4620 to 3959. Then, TP value followed by a quickly descending trend until June 2008. The second cycle started at June 2008 and ended at October 2008, which was in the later period of the subprime crisis, and in the initial period of the European sovereign debt crisis. During this period, the CSI 300 Index dropped to 1948 points (almost 54%) from 3611 to 1663. Thereafter, the TP value entered a stationary period from October 2009 to June 2015. The Third cycle began at May 2015 and ended at August 2015, which was in the period of China’s stock market disaster. At this stage, the CSI 300 Index declined by 1474 points (nearly 31%) from 4840 to 3366. Henceforth, the TP value entered another stationary period.

Furthermore, we employ the index of out degree, clustering coefficient, closeness centrality and leaderrank algorithm to measure the risk contagion range, risk contagion extent, risk contagion efficiency and risk contagion degree in each period, respectively. Then, entropy weight TOPSIS method is adopted to compute the score of SIFIs. We list the top 10 of financial institutions with systematically important score and market capitalization (MC) corresponding to the highest TP in three cycles, and the results are shown in Table 5, Table 6 and Table 7.

Table 5, Table 6 and Table 7 display the top 10 financial institutions ranked by the systematically important score on 25 April 2008, 23 September 2008 and 10 July 2015. We can find that CIB, PAB and PSC are included in the three periods, indicating that large commercial banks and insurances play an important role during the financial crisis. Moreover, the top five largest financial institutions ranked by market capitalization are not all included in the table, and some small financial institutions are also systemically important. It means that the SIFIs in network may be “too big to fail” or “too interconnected to fail”.

## 5. Conclusions and Discussion

This paper adopts 5-min high-frequency data of China’s financial institutions to extract realized jump volatility. Then, we employ Granger-casuality test to construct the jump volatility spillover network. Furthermore, out degree, clustering coefficient, closeness centrality and leaderrank value are used to evaluate the SIFIs, respectively. In addition, we utilize entropy weight TOPSIS to comprehensively evaluate the SIFIs.

Some basic results of our research can be summed up as follows: (1) The highest frequency of jump volatility is 44.30% in 2008. This may be related to the outbreak of the subprime mortgage crisis in 2008. (2) We measure the SIFIs from four dimensions. In terms of risk contagion range, we can find that CMB, BOC and CNCB, which are all from depository sector, possess the highest out degree value of 10. One possible explanation is that China’s financial system is a depository-led system. In terms of risk contagion extent, one can see that SLSC and HTSEC, which are all from broke-dealer sector, have the highest clustering coefficient value. This indicates that broke-dealer sector’s risk contagion ability can not be neglected in China’s financial system. In terms of risk contagion efficiency, we discover that CJSC and PAI have the highest closeness centrality value, which means that some insurances have gradually become important departments. In terms of risk contagion degree, the results reveal that the greatest degree of risk contagion is insurance sector, followed by broke-dealer sector, and depository sector have the worst risk contagion degree. (3) Based on the comprehensive evaluation of the SIFIs, by the method of entropy weight TOPSIS, the obtained results show that CLI, CCB, BOB, ICBC and BOCOM are identified as the influential nodes. (4) According to highest values of total linkages in each period, we can find three prominent cycles. The first cycle started at January 2008 and ended at April 2008, which was in the period of the subprime crisis. The second cycle started at June 2008 and ended at October 2008, which was in the later period of the subprime crisis, and in the initial period of the European sovereign debt crisis. The third cycle began at May 2015 and ended at August 2015, which was in the period of China’s stock market disaster. (5) Total connectedness of financial institution networks reveal that large commercial banks and insurances play an important role when financial market is under pressure, especially during the subprime crisis, the European sovereign debt crisis and China’s stock market disaster. Meanwhile, some small financial institutions are also systemic importance, which may be related to their too much interconnection with other financial institutions.

By the way, the work presented in this article does not consider the following points: (1) The data do not contain all publicly listed financial institutions in China because we have deleted those for which we have experienced long suspension periods. Therefore, developing new tools that have limited sample for investigating SIFIs is a worthy target. (2) We just select 24 top financial institutions. Non-financial institutions may also play an important role as a result of their interactions with these financial ones. It would be interesting to research some financial institutions and non-financial institutions at the same time. We will leave this challenging yet interesting problem as future research. (3) It is important to measure SIFIs based on the network. This paper employs linear Granger-casuality test to construct the jump volatility spillover network. There is a nonlinear relationship between financial markets. The method of network construction could be extended by employing the Granger-casuality test. We have to leave these challenging yet interesting problems as future research.

## Figures and Tables

**Table 1:** The descriptive statistics of 5-min high-frequency closing price data of 24 financial institutions.

Code	Financial Institution	Abbreviation	Mean	SD	MAX	MIN
Panel A: Depositories						
S000001.SZ	Ping An Bank	PAB	14.77	5.38	44.45	8.03
S002142.SZ	Bank of Ningbo	NBCB	13.00	3.58	24.27	5.87
S600000.SH	Shanghai Pudong Development Bank	SPDB	14.73	6.91	61.80	7.11
S600015.SH	Huaxia Bank	HXB	10.37	2.14	23.52	6.14
S600016.SH	China Minsheng Banking Corp., Ltd.	CMBC	7.56	1.85	16.38	3.89
S600036.SH	China Merchants Bank	CMB	17.21	6.57	43.58	9.42
S601009.SH	Bank of Nanjing	NJBK	11.24	3.55	23.49	6.41
S601166.SH	Industrial Bank	CIB	18.94	8.47	61.79	8.62
S601169.SH	Bank of Beijing	BOB	10.24	3.19	22.57	5.53
S601328.SH	Bank of Communications	BOCOM	6.01	1.76	16.15	3.60
S601398.SH	Industrial and Commercial Bank of China Ltd.	ICBC	4.61	0.83	8.35	3.15
S601939.SH	China Construction Bank	CCB	5.39	1.17	10.18	3.50
S601988.SH	Bank of China	BOC	3.53	0.69	6.93	2.44
S601998.SH	China CITIC Bank	CNCB	5.60	1.29	10.93	3.39
Panel B: Broker-dealers						
S000686.SZ	Northeast Securities	NESC	17.82	8.78	56.33	5.10
S000728.SZ	Guoyuan Securities	GYSC	15.36	7.00	45.70	5.55
S000783.SZ	Changjiang Securities	CJSC	11.50	4.78	40.15	4.07
S600030.SH	CITIC Securities	CITICS	19.05	10.57	98.07	9.10
S600109.SH	Sinolink Securities	SLSC	17.83	9.28	71.66	5.77
S600837.SH	Haitong Securities	HTSEC	14.17	7.25	61.58	7.06
S601099.SH	Pacific Securities	PSC	9.66	6.30	47.45	1.94
Panel C: Insurances						
S601318.SH	Ping An Insurance (Group) Co. of China, Ltd.	PAI	47.47	14.37	112.55	19.90
S601601.SH	China Pacific Insurance (Group) Co., Ltd.	CPIC	24.72	7.09	50.25	10.36
S601628.SH	China Life Insurance (Group) Co., Ltd.	CLI	23.24	6.65	60.60	12.89

Note: SZ denotes that financial institution is transacted by the Shenzhen stock exchange market, and SH means that financial institution is transacted by the Shanghai stock exchange market.

**Table 2:** Indicators score of 24 financial institutions.

Symbol	OD	C	CC	LR
PAB	2	1.0000	0.2602	0.0200
NBCB	8	0.8393	0.1667	0.0120
SPDB	8	0.5600	0.1684	0.0389
HXB	9	0.6889	0.1633	0.0151
CMBC	8	0.7632	0.1739	0.0645
CMB	10	0.6574	0.1509	0.0157
NJBK	1	0.0000	0.2883	0.0120
CIB	1	1.0000	0.2909	0.0129
BOB	9	0.5688	0.1739	0.0806
BOCOM	8	0.6081	0.1928	0.0747
ICBC	9	0.5854	0.1633	0.0786
CCB	8	0.5112	0.1882	0.0930
BOC	10	0.6136	0.1538	0.0403
CNCB	10	0.6667	0.1495	0.0120
NESC	1	1.5000	0.2991	0.0157
GYSC	6	0.8429	0.1975	0.0302
CJSC	1	0.0000	0.3107	0.0271
CITICS	3	0.8333	0.2222	0.0157
SLSC	2	2.0000	0.2520	0.0120
HTSEC	2	2.0000	0.2520	0.0120
PSC	4	1.0833	0.2133	0.0120
PAI	1	0.5278	0.3107	0.0764
CPIC	7	0.6685	0.1975	0.0720
CLI	9	0.3382	0.1684	0.1563

**Table 3:** The score of systematic importance of financial institutions.

Rank	Symbol	Score	Rank	Symbol	Score
1	CLI	0.7489	13	GYSC	0.2846
2	CCB	0.5160	14	CMB	0.2829
3	BOB	0.4962	15	CNCB	0.2703
4	ICBC	0.4898	16	HXB	0.2658
5	BOCOM	0.4605	17	NBCB	0.2539
6	CPIC	0.4385	18	NESC	0.2239
7	CMBC	0.4409	19	PSC	0.2088
8	BOC	0.3697	20	PAB	0.1914
9	PAI	0.3244	21	CITICS	0.1701
10	SPDB	0.3179	22	CIB	0.1472
11	SLSC	0.2920	23	CJSC	0.0705
11	HTSEC	0.2920	24	NJBK	0.0123

**Table 4:** The correlation between the market capitalization and four indicators.

Index	Equal Weight	PCA	TOPSIS	EWTOPSIS
Correlation coefficients	0.3355	0.5193 ***	0.4871 ***	0.5647 ***

Note: *** denotes significant at 1%.

**Table 5:** Top 10 financial institutions ranked by the score of SIFIs on 25 April 2008.

Rank	Symbol	Score	Rank of MC
1	PSC	0.9087	18 (46,722,978,887)
2	CIB	0.8685	11 (199,800,000,000)
3	NBCB	0.8603	20 (36,175,000,000)
4	NESC	0.8553	24 (18,917,836,544)
5	HTSEC	0.8381	12 (176,610,181,629)
6	ICBC	0.8361	1 (2,167,782,336,669)
7	BOCOM	0.8336	5 (522,280,130,274)
8	PAB	0.8219	16 (64,100,729,703)
9	GYSC	0.7977	19 (42,005,029,000)
10	SLSC	0.7787	23 (22,425,428,420)

Note: This table lists the market capitalization (MC) and its corresponding rank of top 10 financial institutions.

**Table 6:** Top 10 financial institutions ranked by the score of SIFIs on 23 September 2008.

Rank	Symbol	Score	Rank of MC
1	CIB	0.9579	14 (79,900,000,000)
2	CMB	0.9230	7 (251,933,891,562)
3	PSC	0.9229	18 (27,435,468,619)
4	NBCB	0.9100	21 (18,750,000,000)
5	PAB	0.8718	16 (36,046,919,598)
6	ICBC	0.8123	1 (1,452,981,997,613)
7	CCB	0.8122	2 (1,088,991,131,440)
8	CITICS	0.8014	10 (133,603,922,140)
9	PAI	0.7996	6 (261,557,349,223)
10	BOCOM	0.7904	5 (292,496,470,707)

**Table 7:** Top 10 financial institutions ranked by the score of SIFIs on 10 July 2015.

Rank	Symbol	Score	Rank of MC
1	HTSEC	0.9081	14 (256,372,893,000)
2	PAB	0.8741	15 (212,626,927,426)
3	CJSC	0.8731	19 (62,553,148,673)
4	CIB	0.8451	10 (336,845,313,758)
5	PSC	0.8150	23 (40,635,675,469)
6	HXB	0.7961	16 (143,827,801,960)
7	CMB	0.7886	7 (478,924,867,963)
8	SPDB	0.7878	12 (322,331,986,051)
9	CITICS	0.7845	11 (335,880,700,848)
10	CLI	0.7522	4 (1,019,790,556,400)
